# Melioidosis in Myanmar

**DOI:** 10.3390/tropicalmed3010028

**Published:** 2018-03-01

**Authors:** Mo Mo Win, Elizabeth A. Ashley, Khwar Nyo Zin, Myint Thazin Aung, Myo Maung Maung Swe, Clare L. Ling, François Nosten, Win May Thein, Ni Ni Zaw, May Yee Aung, Kyaw Myo Tun, David A. B. Dance, Frank M. Smithuis

**Affiliations:** 1 Department of Medical Research, Yangon, Myanmar; 2 Myanmar Oxford Clinical Research Unit, Yangon, Myanmar; liz@tropmedres.ac (E.A.A.); myomgswe@gmail.com (M.M.M.S.); frank.m.smithuis@gmail.com (F.M.S.); 3 Centre for Tropical Medicine and Global Health, Nuffield Department of Clinical Medicine, Old Road Campus, University of Oxford, Oxford OX3 7FZ, UK; clare@tropmedres.ac (C.L.L.); francois@tropmedres.ac (F.N.); david.d@tropmedres.ac (D.A.B.D); 4 Department of Microbiology, Yangon General Hospital, University of Medicine 1, Yangon, Myanmar; drknzin@gmail.com; 5 Pathology Department, Microbiology Section, North Okkapala General Hospital, University of Medicine 2, Yangon, Myanmar; myintthazinaung@gmail.com; 6 Shoklo Malaria Research Unit, Mahidol-Oxford Tropical Medicine Research Unit, Faculty of Tropical Medicine, Mahidol University, Mae Sot 63110, Thailand; 7 Mandalay General Hospital, University of Medicine, Mandalay, Myanmar; winmaythein14@gmail.com (W.M.T.); sandblood7@gmail.com (N.N.Z.); 8 Pathology Department, Microbiology section, Thingungyun Hospital, University of Medicine 1, Yangon, Myanmar; immyaung@gmail.com; 9 Department of Preventive & Social Medicine, Defence Services Medical Academy, Yangon, Myanmar; kyaw@dsmrc.net; 10 Lao-Oxford-Mahosot Hospital-Wellcome Trust Research Unit, Microbiology Laboratory, Mahosot Hospital, Vientiane, Laos; 11 Faculty of Infectious and Tropical Diseases, London School of Hygiene and Tropical Medicine, London WC1E 7HT, UK; 12 Medical Action Myanmar, Yangon, Myanmar

**Keywords:** melioidosis, *Burkholderia pseudomallei*, Myanmar

## Abstract

Sporadic cases of melioidosis have been diagnosed in Myanmar since the disease was first described in Yangon in 1911. Published and unpublished cases are summarized here, along with results from environmental and serosurveys. A total of 298 cases have been reported from seven states or regions between 1911 and 2018, with the majority of these occurring before 1949. Findings from soil surveys confirm the presence of *Burkholderia pseudomallei* in the environment in all three regions examined. The true epidemiology of the disease in Myanmar is unknown. Important factors contributing to the current gaps in knowledge are lack of awareness among clinicians and insufficient laboratory diagnostic capacity in many parts of the country. This is likely to have led to substantial under-reporting.

## 1. Introduction and History of Melioidosis in Myanmar

All historical accounts of melioidosis start with Myanmar (formerly Burma), since the disease was first recognized in 1911 in Rangoon (now Yangon) General Hospital by Alfred Whitmore, a British pathologist who worked in Burma between 1906 and 1924 [1]. In 1913, Whitmore reported 38 post-mortem cases of a ‘glanders-like disease’, the majority of whom were male and bore stigmata of morphine injection. Krishnaswamy, Whitmore’s assistant, reported in 1917 that he had personally seen more than 200 cases in Rangoon over six years, during which the disease accounted for approximately one in every 20 autopsies he had conducted [2]. In the ensuing years, reported case numbers in the country dwindled, and after the end of World War II, the disease disappeared from sight for more than 50 years. This hiatus corresponded firstly with the transition from colonial rule to independence (in 1948) and later with a prolonged period of military rule in Myanmar (1962–2011), during which the country became closed off from the rest of the world, investment in healthcare slowed, and research stagnated. Microbiologists working in larger centres inside Myanmar have reported sporadic cases over the last 20 years, but the true epidemiology of the disease is unknown. Most health facilities in Myanmar do not have the capacity to identify *B. pseudomallei*, and most health staff are not aware of this disease. It is therefore likely that melioidosis is significantly underdiagnosed in Myanmar, and the projected annual number of cases (more than 6000) and deaths (more than 3000) from a recent modelling study could well be an accurate reflection of the true situation [3].

## 2. Review of Melioidosis Cases and Presence of *B. pseudomallei* in the Country

We searched the PubMed database for cases of melioidosis or evidence of *B. pseudomallei* using the following search terms: ((Myanmar) OR Burma)) AND ((melioid*) OR pseudomallei) OR glanders*)). A separate search was performed for ‘Parotid abscess AND Myanmar’ (no hits). We also consulted the Myanmar medical literature and the melioidosis global database [4], and contacted consultant microbiologists in the biggest centres in the country (public sector).

### 2.1. Results

Evidence for the presence of *B. pseudomallei* in Myanmar comes from reports of clinical cases, environmental sampling and serosurveys. Melioidosis has never been diagnosed in animals in Myanmar although, by analogy with neighbouring countries, infections in animals are almost certainly occurring undiagnosed.

#### 2.1.1. Clinical Cases

**Figure 1:**
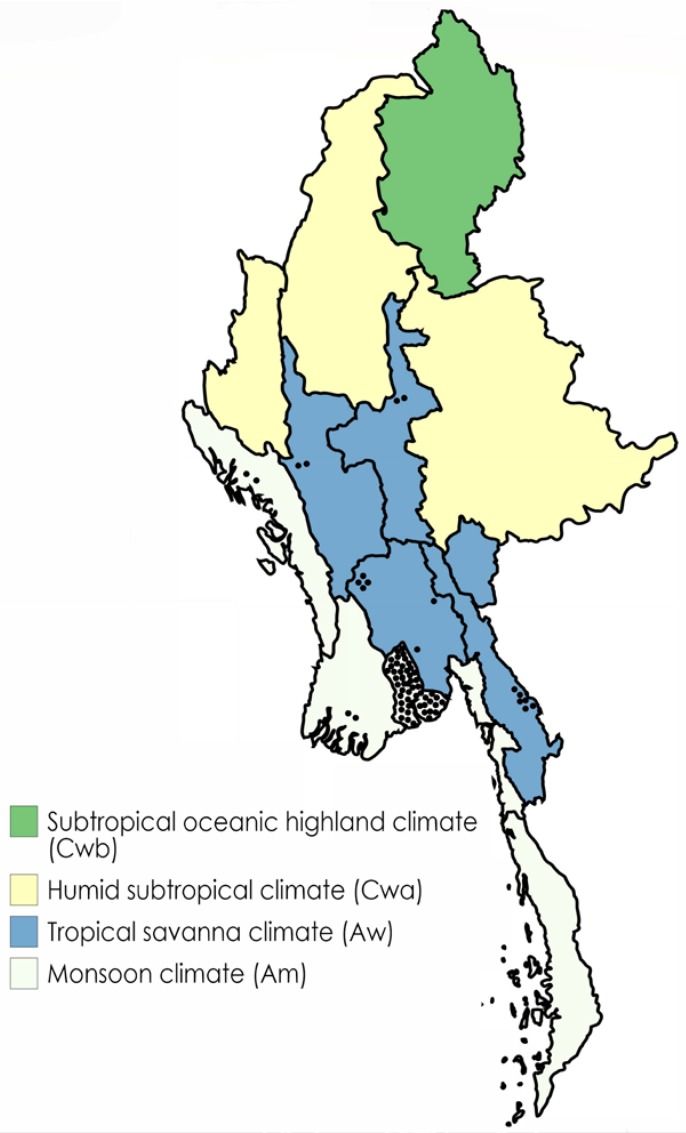
Map of Myanmar showing distribution of reported cases (1912–present) by state or region, categorized by predominant climate type. Each black dot represents a case.

We found a total of 298 published and unpublished cases of melioidosis in Myanmar since 1913 (Table 1). The vast majority (257) were published before the end of 1948. The next report of a case diagnosed inside the country came about half a century later in 2000 in Mandalay [5]. Of the remaining cases, several were diagnosed outside Myanmar, and the remainder came from a very small number of centres. Eighty-five were known to have been culture-confirmed. The report by Krishnaswamy in which he mentions 200 cases does not state explicitly that these were culture-confirmed; however, given his position as Whitmore’s assistant and the descriptions of the first 38 cases in which the diagnosis was confirmed by culture, it is likely that these too were confirmed cases. Of note, only five cases (~2%) were in females and three in children. A total of 276/298 (93%) cases were diagnosed in patients from the Yangon area with six from Kayin state, two from Rakhine state, one from Bago region, two from Ayeyarwady, two from Magway, two from Pyay and four not specified (Table 1 and Figure 1).

In 2014, the results of all the blood cultures processed by the laboratory of Yangon General Hospital between 2005 and 2013 were published [26]. *B. pseudomallei* was not isolated from any of the 3865 cultures; however, the authors note that bacterial isolation and sub-optimal identification techniques may have been one explanation. In addition, the number of cultures requested was low relative to the number of admissions in this busy hospital, which exceeds 50,000 per year.

#### 2.1.2. Serological Evidence for *B. pseudomallei* in Myanmar

The gold standard for the diagnosis of melioidosis is bacterial isolation. Serology is difficult to interpret in endemic countries due to background positivity rates, but gives some indication of population exposure. A cross-sectional sero-survey of 968 migrant workers from Myanmar was undertaken in Thailand, close to the western border, in 2005 using a haemagglutination assay [27]. The majority of participants (99%) came from Kayin or Mon States and Bago or Yangon Regions. The median [IQR] population IHA titre was 1:20 [1:10 to 1:40], with no regional variation observed. Sixty-nine (7%) participants had a titre ≥1:160, which is a recommended cut-off in Thailand used to support a clinical diagnosis of melioidosis. Only 8% of surveyed adults were agricultural workers. Between 2016 and 2017, blood samples were taken for serological testing using a hemolysin co-regulated protein 1 (Hcp1)-based ELISA from 265 febrile patients presenting to a three outpatient clinics in a large suburb of Yangon as part of a research study [28]. All patients tested negative (optical density cut-off 1.16); median [interquartile range] of 0.02 [0.01–0.04] [29].

#### 2.1.3. Evidence from Environmental Surveys

Myanmar has a variable landscape, ranging from the delta region in the south and west, to the drier central plains, and ending at the Himalayan foothills in the north. Using a mathematical modelling approach and taking into account known cases, climate and soil type, Myanmar has been predicted to provide a very suitable environment for the causative bacterium [3]. Results of a farm survey performed in two townships in Yangon (Thanlyin and Hmawbi) were reported in 2016. From 120 soil samples and 12 water samples collected and processed according to standard microbiological guidelines for the detection of *B. pseudomallei,* there were seven positive results (confirmed by molecular methods) [30]. A national survey is underway by the authors, and preliminary testing has detected the bacterium by culture of soil samples from townships in Yangon, Kayin and Mon Regions, which is in agreement with the provenance of some of the reported clinical cases (Table 2). Full results should be available by the end of 2018.

## 3. Current Recommendations and Availability of Measures Against Melioidosis

Currently, there is no requirement to notify cases of melioidosis in the country. The Ministry of Health and Sports has set up a national system for communicable disease surveillance and response. This targets epidemic-prone diseases, specific diseases under national surveillance (DUNS), emerging infectious diseases, climate-related communicable diseases, and vaccine-preventable diseases, but not melioidosis [31]. Under-recognition of the disease is very likely due to a lack of awareness among health staff, under-utilisation of microbiology services by clinicians and inability of many laboratories to identify the organism due to poor availability of necessary reagents, as well as a shortage of microbiologists, particularly in rural areas. There are no standard treatment guidelines for melioidosis, and the few patients identified are managed on a case by case basis.

## 4. Awareness of Melioidosis

With the exception of a few ‘enthusiasts’, melioidosis is almost entirely unknown among healthcare providers. Raising awareness among health staff, clinicians and laboratory staff is therefore the essential prerequisite to identifying melioidosis patients. When diagnostic and treatment services for suspected melioidosis cases are established, raising awareness among the population working in agriculture should follow in high-risk areas. Microbiology in Myanmar is perceived as a diagnostic rather than a clinical service, and there is limited interaction between microbiologists and clinicians, which means opportunities to raise awareness are missed. This was highlighted in the report by Hlaing et al. in 2004 of melioidosis co-infection in a patient with tetanus. A report that *B. pseudomallei* had been isolated was duly sent to the ward, but went unnoticed for several days and was acted upon only when the patient deteriorated.

## 5. Current and Future Challenges

The burden of melioidosis in Myanmar needs to be defined. The evidence suggests that the disease is under-recognised and under-reported. While the majority of cases have been reported from Yangon, evidence from a multicentre abscess survey, routine blood culture surveillance data at a large centre and a recent serosurvey suggests it may not be a leading cause of sepsis in the greater Yangon region, despite the fact that it was clearly an important cause of death in the early 20th century [2,21,26]. Reasons for a possible decline in incidence in areas of Yangon where it was common previously are unclear, although the strong link with injection drug use in the early fatal cases raises the possibility of contamination of the morphine, which is likely to have originated from Upper Burma [32]. An alternative explanation is that this group was relatively immunocompromised and more susceptible to infection following exposure in Yangon itself, and that with increasing urbanization, the risk of exposure has fallen. It is probable that there are unrecognised melioidosis ‘hot-spots’ elsewhere, so accurate data across the whole country are needed. Diabetes mellitus, a well-known risk factor for melioidosis, has moved into the top ten causes of death in the country in the last three years (http://www.healthdata.org/myanmar), which may drive up case numbers in the future. Important contributing factors to the current lack of knowledge are a lack of microbiology laboratory diagnostic capacity, particularly in smaller townships and rural areas, low rates of blood culture requesting, and lack of awareness of the disease among clinicians. Many laboratories at township level have been upgraded as part of the National Health Plan, but are not functional due a shortage of microbiologists. Myanmar has a national reference laboratory (National Health Laboratory), established in 1963, and a system for disease reporting. Addition of *B. pseudomallei* to the list of notifiable pathogens might yield more information on the distribution of cases in the country, but is unlikely to uncover large numbers until the capacity and usage of diagnostic microbiology increase. Environmental surveys may be useful to help target scarce resources to augment case-based surveillance in areas where it is likely to have the highest yield. This should be accompanied by advocacy meetings with state/regional and township health authorities to raise awareness among clinicians and health providers.

## Figures and Tables

**Table 1:** Cases of melioidosis reported from Myanmar.

Year of Report	First Author	N Cases	N Fatalities	N Culture Confirmed	Location in Myanmar	Remarks
1913	Whitmore [1]	38	38	38	Yangon	Case-series identified post-mortem
1915	Knapp [6]	11	11	1 (remaining NS)	Yangon	Case-series identified post-mortem
1917	Krishnaswamy [2]	~200	~200	NS	Yangon	Cases of ‘morphia injector’s septicaemia’ identified post-mortem
1945	Cox [7]	1	1	1	Not known	US Army soldier
1947	Harries [8]	6	2	5	Between Pyay & Yangon (5), Rakhine (1)	West African soldiers serving in Myanmar
1948	Sen [9]	1	1	1	Yangon	Case identified post-mortem
1979	van der Schaaf [10]	1	1	1	Not known	Ex-Royal Netherlands East Indies Army (KNIL) prisoner of war. Post-mortem identification
1994	Wilairatana [11]	1	0	1	Not known	Myanmar national diagnosed in Bangkok
1999	Kunishima [12,13]	1	0	1	Not known	Returned traveler (diagnosed in Japan)
1999	Lee [14]	1	0	1	Yangon	Returned traveler (diagnosed in Taiwan)
2000	May Kyi Aung [5]	1	0	1	Mandalay	Female
2002	Than Than Aye; referenced in [15]	1	NS	NS	Yangon	Cerebral melioidosis (no information)
2002	Leeuwenburgh [16]	1	0	1	Not known	Returned female traveler (diagnosed in Netherlands)
2004	May Kyi Aung [17]	1	1	1	Yangon	-
2004	Mo Mo Win [18]	1	0	1	Yangon (Hmawbi)	-
2004	Su Su Hlaing [19]	1	0	1	Pyay	Concomitant tetanus
2005	Demar [20]	1	0	1	Shwepyitha (Yangon) or Kyunbin(Bago)	Returned traveler (diagnosed in France)
2008	May Kyi Aung [21]	3	NS	3	Yangon	Survey of 133 patients with abscesses in 22 Yangon hospitals
2012	Thae Thae Min [22]	2	0 ^1^	2	Magway	Study of 307 patients hospitalised with infectious diseases
2013	Zaw Than Htun [15]	3	NS	3	Yangon (Hmawbi, Thanlyin), Ayeyarwady (Mawkyun)	Includes one female
2014	Chu [23]	2	2	2	Thai-Myanmar border (close to Kayin state) ^2^	Two fatal cases (one female)
2014	Mar Mar Kyi [24]	1	0	1	Taungoo (Bago)	Healthy student with multiple skin abscesses
2015	Mo Mo Win [4]	2	1 ^3^	2	Yangon	-
2016	Brummaier [25]	1	0	1	Thai-Myanmar border (close to Kayin state) ^2^	Ten-year-old boy with subcutaneous abscesses
2016	Mo Mo Win [4]	2	1	2	Yangon	-
2016–2017	Shoklo Malaria Research Unit [4]	3	0	3	Thai-Myanmar border (close to Kayin state) ^2^	Includes one child
2017	Mo Mo Win (pers.comm)	7	1	7	Rakhine(1), Ayeyarwady (1), Yangon (5)	Includes one female
2017	Ni Ni Zaw (pers.comm.)	1	NS	1	Mandalay	-
2018	Kyaw Myo Tun (pers.comm.)	1	1	1	Yangon	-
2018	Mo Mo Win (pers.comm)	1	0 ^4^	1	Yangon	-
	TOTAL	298				

Cases are adult males unless otherwise stated. ^1^ One patient survived. The other patient was referred to another centre (no outcome recorded). ^2^ The cases from the Thai-Myanmar border were diagnosed in a migrant population from Myanmar. ^3^ Second patient’s outcome not known. ^4^ Discharged home in pre-terminal condition at request of family; NS = not stated.

**Table 2:** Preliminary results of a soil survey being conducted in Myanmar.

Site Number	State or Region	Township	Total Number of Samples	Number of Positive Samples
1	Yangon	Hmawbi^1^	40	0
2	Yangon	Tontay	10	5
3	Yangon	Thanlyin	20	0
4	Yangon	Kyauktan	20	3
5	Yangon	Thone Gwa	20	0
6	Yangon	Kha Yan	20	0
7	Yangon	Dala	20	0
8	Kayin	Kyain Seikgyi	60	0
9	Kayin	Myawaddy	140	9
10	Kayin	Kawkareik	50	1
11	Kayin	Hpa-an	50	1
12	Mon	Kyaikhto	40	0
13	Mon	Bilin	30	0
14	Mon	Thaton	30	0
15	Mon	Yae	30	0
16	Mon	Thanbyuzayat	40	5
17	Mon	Kyaikmaraw	30	2
18	Mon	Mawlamyaing	20	0
19	Mon	Chaung Sone	20	0
20	Mon	Paung	20	0

^1^ Note: an earlier survey found *B. pseudomallei* in Hmawbi (see text).
